# Neuronal ceroid lipofuscinosis in the Russian population: Two novel mutations and the prevalence of heterozygous carriers

**DOI:** 10.1002/mgg3.1228

**Published:** 2020-05-15

**Authors:** Anastasiya A. Kozina, Elena G. Okuneva, Natalia V. Baryshnikova, Olga B. Kondakova, Ekaterina A. Nikolaeva, Inessa D. Fedoniuk, Svetlana V. Mikhailova, Anna Y. Krasnenko, Ivan F. Stetsenko, Nikolay A. Plotnikov, Olesia I. Klimchuk, Yaroslav V. Popov, Ekaterina I. Surkova, Peter A. Shatalov, Alexander S. Rakitko, Valery V. Ilinsky

**Affiliations:** ^1^ Institute of Biomedical Chemistry Moscow Russia; ^2^ Pirogov Russian National Research Medical University Moscow Russia; ^3^ Genotek Ltd. Moscow Russia; ^4^ Scientific and Practical Centre of Pediatric Psychoneurology of Moscow Healthcare Department Moscow Russia; ^5^ Veltischev Research and Clinical Institute for Pediatrics of the Pirogov Russian National Research Medical University Moscow Russia; ^6^ Russian Children’s Clinical Hospital Moscow Russia; ^7^ Faculty of Mechanics and Mathematics Lomonosov Moscow State University Moscow Russia; ^8^ Vavilov Institute of General Genetics Moscow Russia

**Keywords:** exome sequencing, heterozygous carrier, NCL, neuronal ceroid lipofuscinosis

## Abstract

**Background:**

Neuronal ceroid lipofuscinoses (NCLs) are a group of neurodegenerative disorders characterized by an accumulation of lipofuscin in the body's tissues. NCLs are associated with variable age of onset and progressive symptoms including seizures, psychomotor decline, and loss of vision.

**Methods:**

We describe the clinical and molecular characteristics of four Russian patients with NCL (one female and three males, with ages ranging from 4 to 5 years). The clinical features of these patients include cognitive and motor deterioration, seizures, stereotypies, and magnetic resonance imaging signs of brain atrophy. Exome sequencing was performed to identify the genetic variants of patients with NCL. Additionally, we tested 6,396 healthy Russians for NCL alleles.

**Results:**

We identified five distinct mutations in four NCL‐associated genes of which two mutations are novel. These include a novel homozygous frameshift mutation in the *CLN6* gene, a compound heterozygous missense mutation in the *KCTD7* gene, and previously known mutations in *KCTD7*, *TPP1*, and *MFSD8* genes. Furthermore, we estimated the Russian population carrier frequency of pathogenic and likely pathogenic variants in 13 genes associated with different types of NCL.

**Conclusion:**

Our study expands the spectrum of mutations in lipofuscinosis. This is the first study to describe the molecular basis of NCLs in Russia and has profound and numerous clinical implications for diagnosis, genetic counseling, genotype–phenotype correlations, and prognosis.

## Graphical abstract

5 distinct mutations were identified in 4 NCL‐caused genes, of which two mutations are novel. Also carrier frequency of pathogenic and likely pathogenic variants in 13 genes associated with different types of NCL were estimated in Russian population.

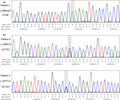

## 1 INTRODUCTION

Neuronal ceroid lipofuscinoses (NCLs) are a group of inherited lisosomal storage diseases with variable age of onset (from early childhood to adulthood). NCL has been recognized as one of the most frequent childhood‐onset neurodegenerative pathologies, with a prevalence of 1:1,000,000 to 1:14,000 worldwide (Haltia & Goebel, 2013). Most NCLs are inherited in an autosomal recessive manner and are clinically characterized by epileptic seizures, psychomotor decline, visual impairment, and premature death. Autosomal dominant inheritance has been reported in one adult‐onset form caused by mutations in *DNAJC5/CLN4* (Online Mendelian Inheritance in Man [OMIM] 611,203; Nosková et al., 2011).

Lysosomal accumulation of autofluorescent lipopigments and proteins in the central nervous system is a key pathological finding of NCLs. These storage granules are stained positively with Luxol fast blue, periodic acid‐Schiff, and Sudan black B methods. However, the structural appearance of inclusion material varies according to each disease type (Haltia, 2006). Biochemical characterization of storage material has identified lipophilic proteins, including subunit C of mitochondrial ATP synthase (major stored protein in most types of NCLs) and sphingolipid activator proteins A and D (main proteins for infantile type of NCL; Fearnley et al., 1990; Palmer, Bayliss, & Westlake, 1995; Palmer et al., 1992; Tyynelä, Baumann, Henseler, Sandhoff, & Haltia, 1995; Tyynelä, Palmer, Baumann, & Haltia, 1993).

Prior to the discovery of the causative genes, the NCLs were classified primarily by age of onset and ultrastructural abnormalities found with electron microscopy: infantile, late‐infantile, juvenile, and adult‐onset forms. The new classification is structured along seven diagnostic axes: (1) affected gene; (2) mutation; (3) biochemical phenotype; (4) clinical phenotype; (5) ultrastructural features; (6) level of functional impairment; and (7) other remarks (additional genetic, environmental, or clinical features; Williams & Mole, 2012). This seven‐axis system is unwieldy for use in clinical practice and some authors have suggested combining axes 1 and 4 for routine use (Mink, Augustine, Adams, Marshall, & Kwon, 2013).

Over the past two decades, more than 430 variants in 13 candidate genes have been identified in the affected patients. These genes encode lysosomal enzymes (*PPT1/CLN1* (OMIM 600,722), *TPP1/CLN2* (OMIM 607,998), *CTSD/CLN10* (OMIM 116,840), *CTSF/CLN13* (OMIM 603,539)), a soluble lysosomal protein (*CLN5* (OMIM 608,102)), a protein in the secretory pathway (*GRN/CLN11* (OMIM 138,945)), two cytoplasmic proteins that also peripherally associate with membranes (*DNAJC5/CLN4* and *KCTD7/CLN14* (OMIM 611,725)), and many transmembrane proteins with different subcellular locations (*CLN3* (OMIM 607,042), *CLN6* (OMIM 606,725), *MFSD8/CLN7* (OMIM 611,124), *CLN8* (OMIM 607,837), and *ATP13A2/CLN12* (OMIM 610,513); Mole & Cotman, 2015). The gene responsible for CLN9 has not been identified. In 2012 Haddad and colleagues reclassified CLN9 as a CLN5 variant (Haddad et al., 2012). Mutations in NCL genes range from those that are described in one or only a few families, to those that are more common in certain populations due to local founder effects. An overview of the different NCL types is given in Table 1.

**TABLE 1:** Associated genes and reported incidence of NCL types

NCL type (OMIM number)	Gene (OMIM number)	Mode of inheritance	Onset	Incidence
CLN1 (256,730)	*PPT1* (600,722)	AR	Infantile NCL (classic), late infantile NCL (variant), juvenile NCL (variant), adult NCL	0.05–5 per 100,000 (Cardona & Rosati, 1995; Claussen et al., 1992; Elleder et al., 1997; Uvebrant & Hagberg, 1997)
†CLN2 (204,500)	*TPP1* (607,998)	AR	Late infantile NCL (classic), juvenile NCL (variant)	0.15–9 per 100,000 (Moore et al., 2008; Taschner et al., 1999; Teixeira et al., 2003)
CLN3 (204,200)	*CLN3* (607,042)	AR	Juvenile NCL (classic)	0.02–4.8 per 100,000 (Elleder et al., 1997; Mitchison et al., 1995; Moore et al., 2008; Teixeira et al., 2003)
CLN4A (204,300)	*CLN 6* (606,725)	AR	Adult NCL	—
CLN4B (162,350)	*DNAJC5* (611,203)	AD	Adult NCL	—
CLN5 (256,731)	*CLN5* (608,102)	AR	Late infantile NCL (variant), juvenile NCL (variant)	0.07 per 100,000 (Santorelli et al., 2013)
†CLN6 (601,780)	*CLN6* (606,725)	AR	Late infantile NCL (variant), adult NCL	0.20–0.62 per 100,000 (Elleder et al., 1997; Santorelli et al., 2013)
†CLN7 (610,951)	*MFSD8* (611,124)	AR	Late infantile NCL (variant)	0.14–2.6 per 100,000 (Moore et al., 2008; Santorelli et al., 2013)
CLN8 (600,143)	*CLN8* (607,837)	AR	Late infantile NCL (variant)	0.07 per 100,000 (Santorelli et al., 2013)
CLN9 (609,055)	—	AR	Late infantile NCL (variant)	
CLN10 (610,127)	*CTSD* (116,840)	AR	Congenital, late infantile NCL (variant), adult NCL	0.01 per 100,000 (Santorelli et al., 2013)
CLN11 (614,706)	*GRN* (138,945)	AR	Adult NCL	—
CLN12 (606,693)	*ATP13A2* (610,513)	AR	Adult NCL	—
CLN13 (615,362)	*CTSF* (603,539)	AR	Adult NCL	—
†CLN14 (611,726)	*KCTD7* (611,725)	AR	Infantile NCL	—

Note

The NCL types found in our study are marked with symbol †. Incidence is the number of new cases of disease in a particular time period.

Abbreviations: AD, autosomal dominant; AR, autosomal recessive; NCL, neuronal ceroid lipofuscinosis; OMIM, Online Mendelian Inheritance in Man.

Here, we describe the clinical and molecular characteristics of four Russian patients with NCL. Exome sequencing and Sanger sequencing were used to analyze the molecular genetics of patients. As most NCL types are recessive diseases, there are also unaffected, heterozygous carriers of the disease. No signs of disease have been associated with being a carrier for NCL. We analyzed genetic data of healthy people to determine carrier frequency for variants of various pathogenicity (according to the ACMG criteria) in 13 genes associated with NCL disease.

## 2 METHODS

### 2.1 Ethical compliance

The study was approved by the ethics committee of Genotek Ltd. (09/2018). Written informed consent was obtained from the patients’ parents for the publication of this report and any accompanying images.

### 2.2 Human subjects

All patients were born in nonconsanguineous families and were normal at birth.

The patients’ clinical symptoms are summarized in Table 2 *.* Skin or rectal biopsies were not performed for any of the patients.

**TABLE 2:** Clinical and molecular phenotype of patients affected by NCL

Patient	Gene	Mutation	State	NCL type	Phenotype	Age, years	Age of onset	Clinical signs
1	*TPP1/CLN2 *(NM_000391.3)	c.622C>T (p.Arg208*)	Homozygote	CLN2	Late infantile NCL (classic)	4	At 2 years	Cognitive and motor deterioration, speech delay, tonic‐clonic seizures, muscular hypotonia, MRI signs of brain atrophy, damage of optic nerves
2	*CLN6* (NM_017882.2)	**c.396dupT (p.Val133fs)**	Homozygote	CLN6	Late infantile NCL (variant)	5	At 3.5 years	Cognitive and motor deterioration, myoclonus, MRI signs of cerebellar cortex atrophy, weight loss
3	*MFSD8/CLN7* (NM_152778.2)	c.525T > A (p.Cys175*)	Homozygote	CLN7	Late infantile NCL (variant)	5	At 2.5 years	Cognitive and motor deterioration, seizures, stereotypies, action myoclonus, partial optic atrophy, MRI signs of cortex, and cerebellar atrophy
4	*KCTD7/CLN14 *(NM_153033.4)	c.190A>G (p.Thr64Ala) **c.337T>C (p.Ser113Pro)**	Compound heterozygote	PME	—	4	At 1 year and 9 months	Cognitive and motor deterioration, ataxia, epileptic paroxysms, and MRI signs of cerebellar subatrophy

Note

Novel mutations are shown in bold.

Abbreviations: NCL, neuronal ceroid lipofuscinosis; PME, progressive myoclonic epilepsy.

#### 2.2.1 Patient 1

The patient presented as a 4‐year‐old boy who was hospitalized with clinical features of motor and cognitive deterioration and seizures. The disease manifested at 2 years of age with attacks of tonic‐clonic seizures with an upward movement of the eyeballs. Wobbly walk and hand tremor developed against a background of anticonvulsant treatment with valproic acid (Depakin). Valproic acid replacement by topiramate (Topamax) was introduced, but without a significant effect. At the age of 3.5 years the patient began to lose previously acquired motor and speech skills. He stopped walking without support and walking with support was accompanied by ataxia.

MRI (magnetic resonance imaging) detected cerebral atrophy and hypoplasia of the cerebellum with atrophic changes. The EEG (electroencephalography) revealed multifocal epileptiform activity in the occipital regions on both sides and in the left centrotemporal region. The electrocardiography showed signs of a sinus‐node dysfunction. Ophthalmologic examination revealed lesions of the visual pathways, mild hypermetropia, and exotropia.

#### 2.2.2 Patient 2

The patient presented as a 5‐year‐old boy who was hospitalized with clinical features of motor and cognitive deterioration, seizures, and weight loss. At the age of 3.5 years he began to lose previously acquired motor and speech skills. At the age of 4.5 years, epileptic seizures appeared in the form of sudden falls with generalized myoclonic twitching for up to 3 s without fainting.

MRI revealed periventricular leukopathy and cortex atrophy of the cerebellar vermis. At the age of 5 years, progressed deterioration was noted: the patient stopped walking and crawling, could only stand with support, and his speech disappeared. The EEG revealed generalized epileptic activity in the form of spike‐wave complexes. Hearing and vision were not impaired.

#### 2.2.3 Patient 3

The patient presented as a 5‐year‐old girl who was hospitalized with clinical features of motor and cognitive deterioration, stereotypies, action myoclonus, epilepsy, and vision loss. For the first 2.5 years the patient was developing according to her age without abnormalities. Starting at the age of 2.5 years, delay in speech and motor development began to progress. At the age of 3 years the first febrile seizure attack occurred. Currently, at the age of 5 years, the patient is only able to roll over and sit with periodic falls.

MRI revealed cortical atrophy, periventricular leukopathy of both hemispheres of the brain, and atrophy of the cerebellum. The EEG revealed a significant delay in the formation of cortical electrogenesis and poorly structured epileptiform activity in the occipital‐parietal‐posterior temporal regions. Ophthalmological evaluation revealed partial atrophy of optic nerves, nystagmus, retinitis pigmentosa, and astigmatism.

Detailed clinical characteristics of Patient 3 were previously described by us (Kozina et al., 2018).

#### 2.2.4 Patient 4

The patient presented as a 4‐year‐old boy who was hospitalized with clinical features of motor and cognitive deterioration and epileptic paroxysms. At the age of 1 year and 9 months he began to fall while walking, and subsequently showed regression of speech development. Worsening of ataxia was noted against a background of anticonvulsant treatment with valproic acid (Depakin, 250 mg twice a day). As a result of hormonal therapy (methylprednisolone), the gait improved and the number of epileptic seizures decreased. Upon examination at 4 years of age, asymmetry of the facial muscles, decreased muscle tone, tendon hyporeflexia, atactic syndrome, unsteady gait, and hand tremor were noted. Speech was characterized by onomatopoeia and a failure to form phrases or sentences.

MRI revealed cerebellar subatrophy and moderate expansion of the lateral ventricles. The EEG detected outbreaks of epileptiform activity. Hearing and vision were not impaired.

### 2.3 Exome sequencing

Exome sequencing was carried out by Genotek Ltd. Genomic DNA from peripheral blood samples was extracted using QIAamp DNA Mini Kit (Qiagen) according to manufacturerʼs protocol. DNA libraries were prepared using NEBNext Ultra DNA Library Prep Kit for Illumina (NEB) according to manufacturer’s protocols. Enrichment was performed with SureSelect XT2 kit (Agilent). Sequencing was performed on HiSeq 2500 (Illumina). After sequencing we trimmed 5′‐ and 3′‐nucleotides with base quality below 20 using Cutadapt (Martin, 2011). Raw reads were aligned to the reference genome build hg19 (GRCh37.p13) using BWA MEM (Li & Durbin, 2009). FastQC was used for data quality control (https://www.bioinformatics.babraham.ac.uk/projects/fastqc/). We called short variants using GATK HaplotypeCaller (McKenna et al., 2010) according to GATK Best Practices for DNA‐seq (DePristo et al., 2011; Van der Auwera et al., 2013). The effect of each mutation was assessed using snpEff (Cingolani et al., 2012). To assess pathogenicity and conservatism, the data were extracted from the dbNSFP (Liu, Wu, Li, & Boerwinkle, 2016), Clinvar (http://www.ncbi.nlm.nih.gov/clinvar/; Landrum et al., 2016), OMIM database (https://omim.org/), and HGMD (http://www.hgmd.cf.ac.uk/ac/index.php). The SIFT (Ng & Henikoff, 2003) and PolyPhen‐2 (http://genetics.bwh.harvard.edu/pph2/; Adzhubei, Jordan, & Sunyaev, 2013) utilities were used to predict pathogenicity of the mutation. Information on the frequency of mutations was taken from the 1,000 Genomes project (http://browser.1000genomes.org/index.html; 1000 Genomes Project Consortium et al., 2015), ExAC (http://exac.broadinstitute.org/; Lek et al., 2016), and Genotek frequency data. Description of mutations and their pathogenicity were predicted according to the Standards and Guidelines developed by ACMG (American College of Medical Genetics and Genomics), Association for Molecular Pathology and College of American Pathologists (Richards et al., 2015).

All variants identified by exome sequencing were confirmed by Sanger sequencing.

### 2.4 Analysis of NCL carrier frequency

To estimate the number of NCL carriers in the Russian population, we used 6,396 samples from the Genotek Ltd database. The average age was 35.4 ± 14.3 years. The group included 49% healthy female and 51% healthy male samples. Data used in our analysis were collected prior to November 2019.

All samples were genotyped using Illumina Global Screening Array v.1 and v.2.

The pathogenicity status of each mutation was assessed according to the ACMG criteria and ClinVar database information. After excluding variants with a frequency of 1% and more, the remaining variants were classified into three classes: pathogenic, likely pathogenic, and uncertain significance.

Carrier frequencies were calculated by dividing the number of heterozygous individuals by the total number of individuals genotyped. Incidence was estimated using the Hardy‐Weinberg principle.

## 3 RESULTS

Using exome sequencing we identified five mutations in four patients with clinical signs of NCL (Table 2). Of the mutations characterized here, two were missense, one created a frameshift, and two created a premature stop codon. All mutations were confirmed by Sanger sequencing.

### 3.1 Patient 1

A homozygous mutation in exon 6 (c.622C>T, p.Arg208*) of the *TPP1* gene (NM_000391.3) was identified in Patient 1. This variant was interpreted as pathogenic according to the variant guidelines of the ACMG (PVS1, PM1, PP3, and PP5). This mutation was described as pathogenic in several patients with late infantile NCL (Barisić, Logan, Pikija, Skarpa, & Blau, 2003; Sleat et al., 1999). This is one of the two most common mutations in *CLN2.*

The classic phenotype of CLN2 disease is one of the two most common forms of NCL, with CLN3 disease being the other, that are characterized by late‐infantile onset (about 2–4 years old). It generally manifests with new‐onset seizures and/or ataxia, typically in combination with a history of early language delay (Fietz et al., 2016).

### 3.2 Patient 2

**FIGURE 1:**
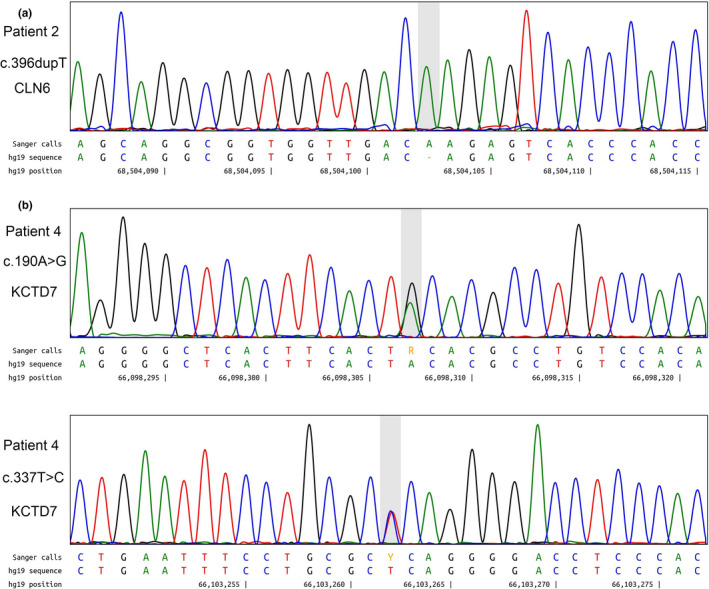
Chromatograms of novel mutations identified in the study. (a) Patient 2 was homozygous for *CLN6* (NM_017882.2) mutation c.396dupT (p.Val133fs). (b) Patient 4 was compound heterozygous for *KCTD7* (NM_153033.4) mutations c.190A>G (p.Thr64Ala) and c.337T>C (p.Ser113Pro). The first mutation was previously reported, the second mutation is novel. R and Y are IUPAC ambiguity codes that mean A+G and C+T, respectively

A novel homozygous mutation in exon 4 (c.396dupT, p.Val133fs) of the *CLN6* gene (NM_017882.2) was identified in Patient 2 (Figure 1). This variant was interpreted as likely pathogenic according to the variant guidelines of the ACMG (PVS1, PM2). This mutation was not reported in 60,706 subjects in ExAC or in 2,535 subjects in the 1,000 Genomes Browser.

The c.396dupT mutation is a duplication in exon 4 that shifted the reading frame after Ser132, extending the protein by 17 extra amino acids. Wheeler and colleagues described a 2‐bp deletion (c.395_397delCT) which leads to a frameshift after Asp131 that also results in 17 extra amino acids (Wheeler et al., 2002).

The *CLN6* gene is predicted to encode a 311–amino acid protein with N‐terminal cytoplasmic domain, seven putative transmembrane domains, and a luminal C‐terminus (Wheeler et al., 2002). As a result of the c.396dupT mutation, four helices and two large extracellular domains are deleted.

Mutations in the *CLN6* gene cause variant late infantile NCL. The age of onset of CLN6 is between 18 months and 8 years and the leading symptoms are motor delay, dysarthria, and ataxia. In approximately 65% of patients, seizures start before the age of 5 years. Visual failure occurs early in 50% of patients. Deterioration is rapid after diagnosis and most children die between the ages of 5 and 12 years (Jalanko & Braulke, 2009).

### 3.3 Patient 3

A homozygous mutation in exon 6 (c.525T>A, p.Cys175*) of the *MFSD8* gene (NM_152778.2) was identified in Patient 3. This variant leads to a premature stop codon and was interpreted as pathogenic according to the variant guidelines of the ACMG (PVS1, PM2, PP3, and PP5). This mutation was not reported in 60,706 subjects in ExAC or in 2,535 subjects in the 1,000 Genomes Browser. We previously described this novel variant (Kozina et al., 2018).

Homozygous or compound heterozygous mutations in the *MFSD8* gene are associated with variant late infantile NCL called the CLN7 disease (OMIM 610,951). This disease usually begins with seizures and a loss of acquired skills. The disorder is progressive, with mental regression, speech impairment, and loss of vision (Aiello et al., 2009).

### 3.4 Patient 4

Two heterozygous mutations in exon 2 (c.190A>G, p.Thr64Ala) and exon 3 (c.337T>C, p.Ser113Pro) of the *KCTD7* gene (NM_153033.4) were identified in Patient 4 (Figure 1).

The c.190A>G mutation has been described as a mutation with conflicting interpretations of pathogenicity in ClinVar (https://www.ncbi.nlm.nih.gov/clinvar/variation/195417/). This variant was interpreted as having uncertain significance according to the variant guidelines of the ACMG (PM2, PP2, and PP3). This mutation was predicted to be pathogenic by SIFT (score 0.00) and Polyphen2 (HumDiv score 1.00; HumVar score 0.99).

The c.337T>C mutation is novel. This variant was interpreted as having uncertain significance according to the variant guidelines of the ACMG (PM2, PP2, and PP3). This mutation was predicted to be pathogenic by SIFT (score 0.01) and nonpathogenic by Polyphen2 (HumDiv score 0.62; HumVar score 0.52). This mutation was not reported in 60,706 subjects in ExAC or in 2,535 subjects in the 1,000 Genomes Browser, and was not found in the Genotek exome database.

The first mutation (c.190A>G) is present in the father of proband and the second mutation (c.337T>C) is present in the mother, which confirms the compound‐heterozygous localization of these two mutations.

We also identified a previously reported variant in the *SCN9A* gene (NM_002977.3, c.3316G>T, p.Val1106Leu) inherited from the father. This variant was interpreted and described in the databases as having uncertain significance according to the variant guidelines of the ACMG (PP3 and BP1). This mutation was predicted to be pathogenic by SIFT (score 0.00) and nonpathogenic by Polyphen2 (HumDiv score 0.00; HumVar score 0.01). The clinical symptoms of the father do not include seizures.

Homozygous mutations in the *KCTD7* gene were previously reported to cause infantile NCL called the CLN14 disease (OMIM 611,726; Staropoli et al., 2012). Most homozygous and compound heterozygous mutations in the *KCTD7* have been described in patients with progressive myoclonic epilepsy (PME) who have normal intracellular accumulation of storage material (Staropoli et al., 2012). Patient 4 was diagnosed with PME because the analysis of storage material was not carried out for the patient.

Mutations in the *SCN9A* gene which encodes the α‐subunit of the voltage‐gated sodium channel, Nav1.7, were found to be responsible for febrile seizures. In our case the *SCN9A* mutation is likely not the primary disease‐causing mutation, but may have modified the phenotype. Singh and colleagues presented preliminary evidence that a mutation in the *SCN9A* gene may act as a genetic modifier of Dravet syndrome when found in conjunction with an *SCN1A* mutation (Singh et al., 2009).

We also revealed two other patients with heterozygous variants in the *DNAJC5* gene and one patient with compound heterozygous variants in the *ATP13A2* gene.

Variants in the *DNAJC5* (NM_025219.2) gene were heterozygous missense mutations in exon 3 (c.217A>G, p.Arg73Gly) and exon 5 (c.538G>A, p.Glu180Lys).

The first variant (c.217A>G, p.Arg73Gly) was discovered in a 14‐year‐old girl with LQT syndrome. This variant was interpreted as having uncertain significance according to the variant guidelines of the ACMG (PM2, PP3). This variant was predicted to be pathogenic by SIFT (score 0.00) and Polyphen2 (HumDiv score 0.98; HumVar score 0.94). The total population frequency of this variant is 0%. This variant was not reported in ClinVar.

The second variant (c.538G>A, p.Glu180Lys) was discovered in a 9‐year‐old girl with motor and cognitive deterioration, congenital developmental anomalies of the hands, and cleft palate. This variant was interpreted as having uncertain significance according to the variant guidelines of the ACMG (PM2, PP3). This variant was predicted to be likely pathogenic by SIFT (score 0.05) and pathogenic/nonpathogenic by Polyphen2 (HumDiv score 0.97; HumVar score 0.54). The total population frequency of this variant remains unknown. This variant was not reported in ClinVar.

Two NCL‐causing mutations in the *DNAJC5* gene were previously described in the literature. These mutations are located in the region that encodes the cysteine‐string domain of the protein (Nosková et al., 2011). None of the mutations that were identified by us are located near the cysteine‐string domains. Neither of our two patients with *DNAJC5* mutations had signs of NCL at the time of the examination. However, as the disease is rare, a connection with the gene was only established recently, and the disease has a late onset, we cannot accurately determine the clinical significance of the identified variants. Two variants in the *ATP13A2* (NM_022089.3) gene were discovered in a 7‐year‐old boy with Charcot‐Marie‐Tooth disease: c.59C>T (p.Thr20Met) in exon 2 and c.533C>T (p.Thr178Ile) in exon 6. The first variant (c.59C>T) was interpreted as likely benign according to the variant guidelines of the ACMG (PM2, BP1, and BP4). This variant was predicted to be likely pathogenic by SIFT (score 0.09) and nonpathogenic by Polyphen2 (HumDiv score 0.14; HumVar score 0.01). The total population frequency of this variant is 0%. This variant was not reported in ClinVar. The second variant (c.533C>T) was interpreted as having uncertain significance according to the variant guidelines of the ACMG (PM2 and BP1). This variant was predicted to be likely pathogenic by SIFT (score 0.09) and nonpathogenic by Polyphen2 (HumDiv score 0.39; HumVar score 0.07). The total population frequency of this variant remains unknown. This variant was not reported in ClinVar. Homozygous or compound heterozygous mutations in the *ATP13A2* gene lead to the development of the CLN12 (Kufor‐Rakeb syndrome) with juvenile onset. Biallelic mutation in the *ATP13A2* gene also causes autosomal recessive spastic paraplegia‐78 (OMIM 617,225) with adult onset. Due to the later onset of both diseases, we cannot predict whether these mutations will lead to the disease. This patient had typical signs of the Charcot‐Marie‐Tooth disease: gait abnormality, ENMG signs of axonal motor‐sensory damage, and a delay in skill development. Exome sequencing revealed a likely pathogenic mutation in the *PMP22* gene as a probable cause of the Charcot‐Marie‐Tooth disease.

**FIGURE 2:**
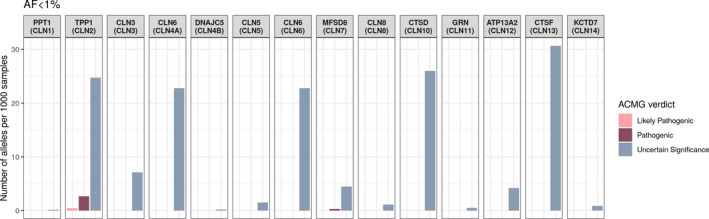
The number of heterozygous carriers with variants in NCL‐associated genes in the Russian population. The analysis presents variants for which the allele frequency is <1%. The pathogenicity status of the mutation was assessed according to the ACMG criteria and ClinVar database. ACMG, American College of Medical Genetics and Genomics; NCL, neuronal ceroid lipofuscinosis

We also estimated the number of heterozygous carriers with variants in 13 NCL‐caused genes in the Russian population (Figure 2).

Pathogenic mutations were observed in the *TPP1* and *MFSD8* genes. The c.622C>T (p.Arg208*) mutation that we found in Patient 1 is one of the two most common mutations in the *TPP1* gene. We found 16 heterozygous carriers of this mutation among 6,396 Russians (estimated frequency 2.5 per 1,000). The largest number of mutations with uncertain significance was found in the *TPP1*, *CLN6*, *CTSD*, and *CTSF* genes. A list of all identified variants and their allele frequencies are shown in Supporting Information.

For two NCL types we estimated the frequency of pathogenic and likely pathogenic mutation carriers in the Russian population (Table 3). We analyzed 6,396 healthy people and found that none of them carried both variants simultaneously.

**TABLE 3:** Carrier frequency for pathogenic and likely pathogenic mutations in two NCL‐caused genes in the Russian population

Gene/NCL type	Carrier frequency (this study)	Estimated incidence (this study)	Incidence (from other studies)
*TPP1*/CLN2	0.0031 (1 in 320)	0.24 per 100,000	0.15–9 per 100,000 (Moore et al., 2008; Taschner et al., 1999; Teixeira et al., 2003)
*MFSD8*/CLN7	0.000313 (1 in 3,198)	0.024 per 100,000	0.14–2.6 per 100,000 (Moore et al., 2008; Santorelli et al., 2013)

Note

The methods for calculating carrier frequency and estimated incidence are given in Section 2.

Abbreviation: NCL, neuronal ceroid lipofuscinosis.

## 4 DISCUSSION

The few available prevalence and incidence studies for NCL types were carried out in small geographic areas, mainly in Europe (Cardona & Rosati, 1995; Claussen, Heim, Knispel, Goebel, & Kohlschütter, 1992; Elleder et al., 1997; Mitchison et al., 1995; Moore et al., 2008; Santorelli et al., 2013; Taschner, Franken, Berkel, & Breuning, 1999; Teixeira et al., 2003; Uvebrant & Hagberg, 1997). CLN2 and CLN3 diseases are the most common types of NCL. However, NCL incidence is known to be dependent on ethnicity. There are a number of estimates for the incidence of NCLs as a collective group in different populations, and these range from ∼0.6 (Italy) to ∼14 (Newfoundland) per 100,000 live births (Sleat, Gedvilaite, Zhang, Lobel, & Xing, 2016). NCLs are more common in Finland, where approximately 1 in 12,500 individuals are affected. Using ExAC data, Sleat and colleagues obtained population frequencies for mutations in different ethnic populations in 12 genes most commonly associated with NCL disease (Sleat et al., 2016). However, the prevalence of NCL heterozygotes in the Russian population was not previously determined.

According to our data, CLN2 and CLN7 are the most common NCL types in the Russian population. Carrier frequency for these types is 1/320 and 1/3,198, respectively. As there are no asymptomatic forms of lipofuscinosis, carrier frequencies can be directly translated into incidence and prevalence. Estimated incidence for CLN2 and CLN7 is 0.24 and 0.024 per 100,000, respectively. Thus, different genes should be prioritized in clinical analysis of potential NCL cases depending on the patient's ancestry.

NCL disorders are marked by considerable clinical and genetic overlap with other congenital neurodegenerative diseases which can lead to delays and difficulties in diagnosing the condition. Our study may help to establish appropriate genetic counseling and prenatal diagnosis for individuals who are at high risk of NCL forms. Furthermore, clinical trials for potential therapies of NCL are currently in progress (https://clinicaltrials.gov/ct2/results?cond=Lipofuscinosis%2C+Neuronal+Ceroid&term=&cntry=&state=&city=&dist=), therefore, knowledge about the prevalence of the disease becomes even more important.

This is the first genetic study from Russia to describe the molecular defects underlying the NCL phenotypes. We identified five pathogenic mutations (including two that are novel) in four affected patients. Two novel mutations were revealed in *CLN6* and *KCTD7* genes. These genes are associated with CLN6 and CLN14, respectively. The *CLN6* gene is also associated with adult‐onset type A Kufs disease. CLN6 is variant late infantile form of NCL with disease onset between 18 months and 8 years of age. This variant exhibits an intermediate phenotype between late infantile NCL and juvenile NCL. CLN14 is an infantile form of NCL with disease onset before 2 years of age (Staropoli et al., 2012).

We did not find the patients with the most common 1‐kb *CLN3* deletion in our database. Due to high prevalence, this mutation is more often detected by other methods before exome sequencing. Our evaluation of carrier frequencies did not include an analysis of 1‐kb *CLN3* deletion due to the limitation of the method.

Our results highlight next‐generation sequencing as a novel and powerful methodology for rapid molecular diagnosis of NCL.

## CONFLICT OF INTEREST

AAK, EGO, NVB, AYK, IFS, NAP, OIK, YVP, EIS, PAS, ASR, and VVI are employees of Genotek Ltd. The authors declare that they have no other competing interests.

## AUTHOR CONTRIBUTIONS

AAK, EGO, NVB, OBK, EAN, IDF, SVM, AYK, IFS, NAP, OIK, YVP, EIS, PAS, ASR, and VVI met the International Committee of Medical Journal Editors (ICMJE) criteria for authorship. AAK, EGO, NVB, OBK, EAN, IDF, SVM, EIS, and PAS coordinated the management and recruitment of patients involved in the study. AAK, EGO, NAP, and EIS contributed to data collection and the first draft of the manuscript. AYK, IFS, NAP, OIK, YVP, and ASR carried out the mutation analysis, interpretation of data, and revised the manuscript. ASR and VVI were the mentors who developed design of the study and substantially revised the manuscript. All authors read and approved the final manuscript.

## ETHICS APPROVAL AND CONSENT TO PARTICIPATE

All research was approved by the ethics committee of Genotek Ltd. (09/2018). The patients’ parents have provided written informed consent.

## CONSENT FOR PUBLICATION

The patients’ parents gave written informed consent to studies and publication of clinical information, images, and sequencing data.

## Supporting information

Supplementary Material

## Data Availability

We did not use new software, databases, or applications/tools in the manuscript, and our data are described in the manuscript, figures, and tables. The data that support the findings of this study are available from the corresponding author upon reasonable request.
